# Unique nucleolar dominance patterns in distant hybrid lineage derived from *Megalobrama Amblycephala* × *Culter Alburnus*

**DOI:** 10.1186/s12863-016-0457-3

**Published:** 2016-12-05

**Authors:** Jun Xiao, Fangzhou Hu, Kaikun Luo, Wuhui Li, Shaojun Liu

**Affiliations:** Key Laboratory of Protein Chemistry and Fish Developmental Biology of the Education Ministry of China, College of Life Sciences, Hunan Normal University, Changsha, 410081 People’s Republic of China

**Keywords:** Nucleolar dominance pattern, Distant hybrid lineage, 45S rRNA gene, SNP

## Abstract

**Background:**

Nucleolar dominance is an epigenetic phenomenon that occurs in interspecific hybrids and involves the expression of 45S rRNA genes inherited from one progenitor due to the silencing of the other progenitor’s rRNA genes. In this paper, changes in the genetics and expression of 45S rRNA genes in F1 and F2 hybrid progeny of blunt snout bream (BSB, *Megalobrama amblycephala*) × topmouth culter (TC, *Culter alburnus*) are investigated.

**Results:**

The 45S rDNA loci were analyzed by cloning, RT-PCR and sequencing methods. The results show that nucleolar dominance patterns differ in the F1 and F2 hybrids. In the F1 hybrids of BSB × TC, all the tested individuals inherited and expressed the 45S rRNA genes of both BSB and TC, indicating that nucleolar dominance is not established in the F1 hybrids. However, in the F2 hybrids of BSB × TC, five patterns are observed. Pattern 1 inherits and expresses only the 45S rRNA gene of BSB. Pattern 2 inherits the 45S rRNA gene from both BSB and TC, but only expresses the 45S rRNA of BSB. Pattern 3 inherits and expresses the 45S rRNA gene from both BSB and TC. Pattern 4 inherits the 45S rRNA gene from both BSB and TC, but only expresses the 45S rRNA gene of TC. Pattern 5 inherits and expresses only the 45S rRNA gene of TC.

**Conclusions:**

Nucleolar dominance shows distinctive patterns in intergeneric hybrids of BSB × TC. It is not established in F1 hybrids and is random in F2 hybrids. This study provides new insights into the phenomenon of nucleolar dominance in genetic hybrids in vertebrates.

**Electronic supplementary material:**

The online version of this article (doi:10.1186/s12863-016-0457-3) contains supplementary material, which is available to authorized users.

## Background

In eukaryotes, 45S rRNA genes are tandemly arrayed by the hundreds, and sometimes by the thousands, at chromosomal loci spanning millions of base pairs. rRNA genes are transcribed by RNA polymerase I (Pol I) into a precursor RNA (prerRNA) that encodes the three largest RNA components of ribosomes [1, 2]. Nucleolar dominance is an epigenetic phenomenon that occurs in genetic hybrids and involves expression of the 45S rRNA genes inherited from only one progenitor. It has been revealed that nucleolar dominance is caused by the silencing of the other progenitor’s rRNA genes [3]. Ribosomal RNA gene silencing involves changes in DNA methylation and histone modifications [4–8]. But the molecular basis for the determination of which progenitor’s genes are silenced remains unclear. Nucleolar dominance is first described in plants [9] and is widely observed in interspecific hybrids of plants, invertebrates, frogs, flies, fish and mammals [10, 11]. However, it has rarely been reported in different generations of hybrid lineages at the level of intergeneric hybridization in vertebrates because the formation of distant hybrid lineages in animals is challenging in practice.

The genetics and expression of 45S rRNA genes have been studied by detecting single nucleotide polymorphism (SNP) sites in the 18S rRNA gene [12]. DNA sequencing, while relatively laborious, is the gold standard in SNP discovery. The most widely used approach is direct DNA sequencing of polymerase chain reaction (PCR) products with dye-terminator chemistry analyzed on automated DNA sequencers [13]. SNPs are easily detected because two peaks are observed in sequence traces in the heterozygous state, compared with a single peak in the homozygous state.

In this study, based on the formation of a bisexual fertile hybrid lineage of blunt snout bream (BSB, *Megalobrama amblycephala*) × topmouth culter (TC, *culter alburnus*) [14], we analyze the genetics and expression patterns of 45S rRNA genes in F1 and F2 hybrids. The results show that the genetics and expression patterns of 45S rRNA genes in the liver of mature individuals differ in F1 and F2 hybrids. Furthermore, the parental 45S rDNA to be silenced appears to be chosen randomly in the F2 hybrids of BSB × TC, contrary to previous reports [3, 15, 16]. This study provides new insights into the phenomenon of nucleolar dominance in genetic hybrids in vertebrates. The hybrid lineages derived from BSB × TC provide good material with which to study the mechanism of nucleolar dominance.

## Results

### ITS1 sequence in parental species and hybrids

**Fig. 1:**
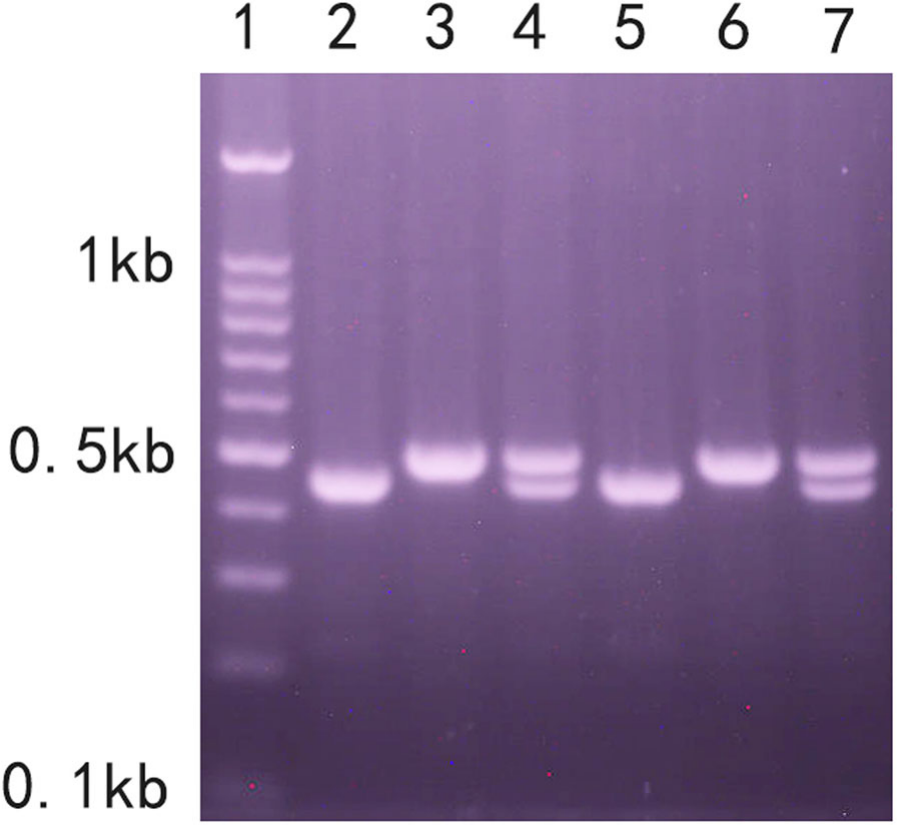
The genotype of ITS1 in the four kinds of fish. Channel 1: 0.1 kb DNA grad mark. Channel 2: PCR results of ITS1 in BSB. Channel 3: PCR results of ITS1 in TC. Channel 4: PCR results of ITS1 in F_1_ hybrids of BSB × TC. Channel 5–7: PCR results of ITS1in F_2_ hybrids of BSB × TC

The PCR results of ITS1 sequences in BSB, TC and F1 and F2 hybrids of BSB × TC are shown in Fig. 1 (for every pattern of each kind of fish, one sample was used to present). In BSB, the ITS1 sequence is homogeneous and 322 bp in length (DDBJ, accession number: AB872813) and similarly in TC the ITS1 sequence is homogeneous but 366 bp in length (DDBJ, accession number: AB872814). The lengths of the fragments (including partial 18S rDNA, complete ITS1 rDNA and partial 5.8S rDNA sequences) amplified by primers P4F/P4A in BSB (441 bp) and TC (487 bp) are clearly differentiated by electrophoresis in 2% agarose gel. Therefore, the fragments can be used as a simple genetic marker to examine the genetic heterogeneity of hybrid progeny of BSB and TC. As shown in Fig. 1, only one kind of ITS1 genotype is found in the F1 hybrids, in which both parents’ ITS1 bands are detected (Fig. 1). However, three kinds of genotypes are found in the F2 hybrids. Some individuals inherit the single ITS1 genotype of either BSB or TC, while others inherit both genotypes.

### Sequence and variation of 18S rRNA genes in parental species

Sequence divergence between 18S rDNA fragments amplified from BSB and TC with the 18S P1F/P1R primer pair is low. We examined intra- and interspecific sequence divergence for 60 sequences from 30 BSB individuals and 60 sequences from 30 TC individuals, consisting of 1565 bp of the 18S rRNA gene. These sequences show 100% similarity between BSB and TC except for a single SNP at position 40, which is fixed for cytosine in BSB and thymine in TC (Additional file 1).

### Expression of 45S rRNA

**Fig. 2:**
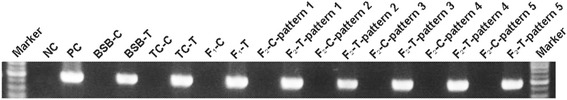
PCR results of cDNA and controls. Gel electrophoresis map of the PCR results of cDNA from BSB, TC, F_1_ hybrids of BSB × TC and F_2_ hybrids of BSB × TC. NC: negative control, PC: positive control. BSB-C: control in the BSB, BSB-T: treatment in the BSB. F_1_-C: the control in F_1_ hybrids of BSB × TC, F_1_-T, the treatment in F_1_ hybrids of BSB × TC. F_2_-C-pattern 1: the control of pattern 1 in F_2_ hybrids of BSB × TC, F_2_-T-pattern 1: the treatment of pattern 1 in F_2_ hybrids of BSB × TC. The other samples are named as the same rules. Note: For every pattern of each kind of fish, one sample was used to present

The expression of 18S rRNA was examined in BSB, TC and F1 and F2 hybrids of BSB × TC using genomic DNA from BSB as template in the PCR positive control and ddH_2_O as template in the PCR negative control. Thirty samples were tested from each kind of fish (BSB, TC, F_1_ and F_2_). Figure 2 shows the 18S rRNA expression in each kind of fish (for every pattern of each kind of fish, one sample was used to present). After digestion with DNase I (Invitrogen), the PCR controls showed that the RNA was free of DNA contamination.

**Fig. 3:**
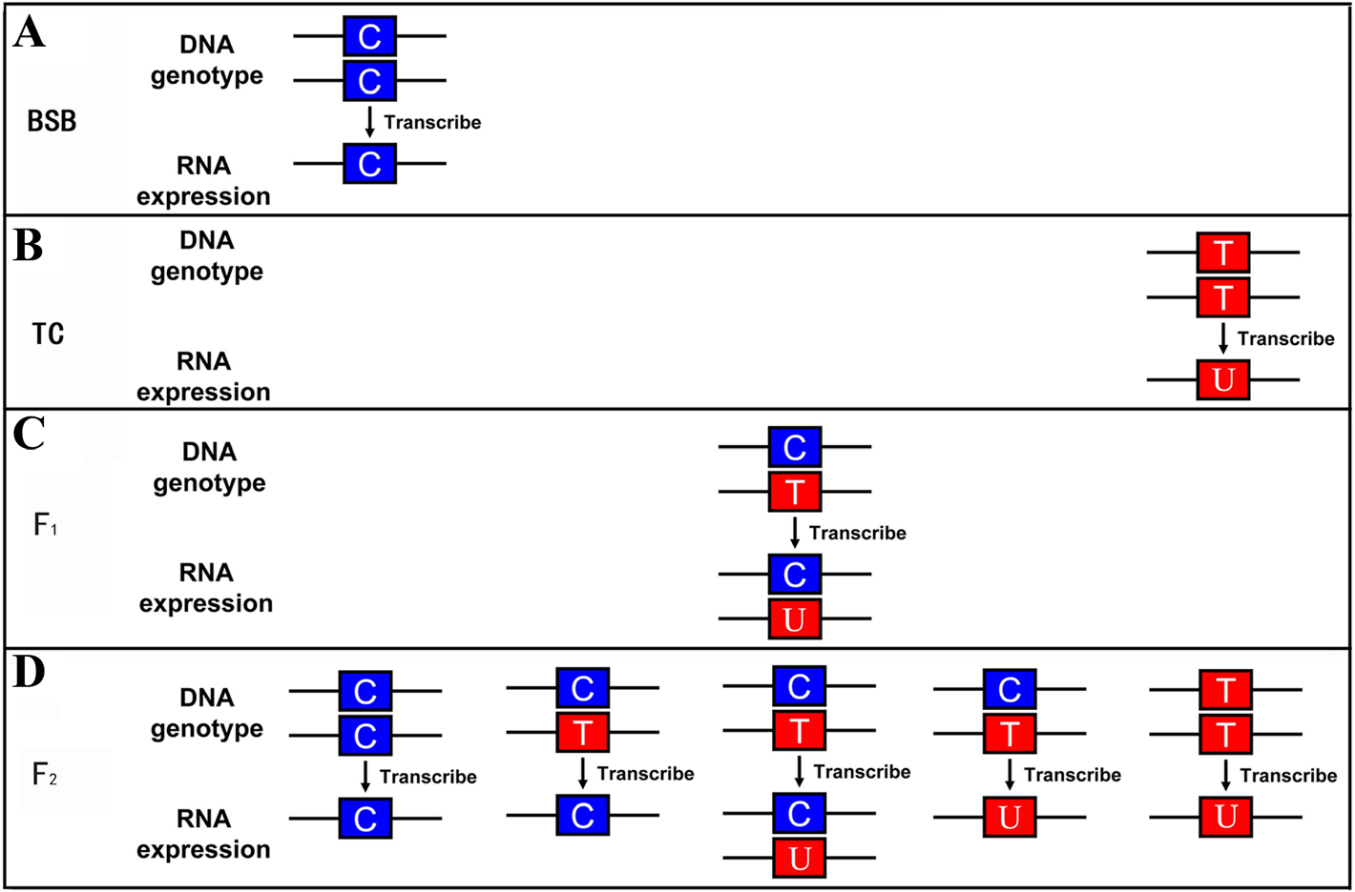
The diagrammatic drawing of genotype and expression of 45S rRNA gene. **a**, **b** The genotype and expression of 45S rRNA gene in BSB. The genotype and expression of 45S rRNA gene in TC. **c** The genotype and expression of 45S rRNA gene in F_1_ hybrids of BSB × TC. **d** The genotype and expression of 45S rRNA gene in F_2_ hybrids of BSB × TC. Note: For every pattern of each kind of fish, one sample was used to present

In the sequencing peak figures, homozygous samples have sequence traces with one peak, whereas heterozygous samples show two peaks. The two patterns are easily distinguishable. In the sequence traces of PCR products amplified with the primer pair 18S P2F/P2R, only one peak is observed at the C308T SNP locus (the same SNP at position 40 of the 18S rDNA amplified by the 18S P1F/P1R primer pair) in genomic DNA and cDNA of the 18S rRNA gene for both BSB and TC (Additional file 2A). In the F1 hybrids analyzed, the genotype for the C308T SNP is heterozygous, and expression of 18S rRNA is also heterozygous with two peaks observed in the sequence traces for PCR products amplified from the cDNA of 18S rRNA (Additional file 1B). Among the F2 hybrids, five patterns are observed. Pattern 1 inherits and expresses only the 45S rRNA gene of BSB (Additional file 1C). Pattern 2 inherits the 45S rRNA gene from both BSB and TC, but only expresses the 45S rRNA of BSB. Pattern 3 inherits and expresses the 45S rRNA gene from both BSB and TC. Pattern 4 inherits the 45S rRNA gene from both BSB and TC, but only expresses the 45S rRNA gene of TC. Pattern 5 inherits and expresses only the 45S rRNA gene of TC. A diagram of the genotype and expression patterns of 45S rRNA genes in BSB and TC and their hybrids is shown in Fig. 3. The frequency distribution of the five different genetic expression patterns of 45S rRNA gene in the F2 hybrids was showed in Table 1.

**Table 1:** The frequency distribution of the 5 different genetic expression patterns of 45S rRNA gene in the F_2_ hybrids

	Number	Percent
Pattern1^a^	3	10%
Pattern2^a^	7	23.3%
Pattern3^a^	13	43.4%
Pattern4^a^	3	10%
Pattern5^a^	4	13.3%
Total	30	100%

^a^ *Note:* Pattern 1 inherits and expresses the single 45S rRNA gene of BSB. Pattern 2 inherits both the 45S rRNA gene from BSB and TC, but only expresses the 45S rRNA of BSB. Pattern 3 inherits and expresses both the 45S rRNA gene from BSB and TC. Pattern 4 inherits both the 45S rRNA gene from BSB and TC, but only expresses the 45S rRNA gene of TC. Pattern 5 inherits and expresses the single 45S rRNA gene of TC

## Discussion

Nucleolar dominance is a phenomenon observed in cells of interspecific hybrids in which nucleolus organizer regions (NORs) derived from one parental species are dominant over those derived from the other parent. Nucleolar dominance was first observed in hybrids of the plant genus *Crepis*, in which the NORs derived from only one species formed secondary constrictions [9]. In subsequent decades, nucleolar dominance has been described in hybrids of many plant genera, including *Brassica* [4] and *Arabidopsis* [17]. A similar phenomenon occurs in hybrids of both *Drosophila* hybrids [18] and *Xenopus* hybrids, in which it has been confirmed that differential rRNA gene transcription is the basis of nucleolar dominance [19]. A form of nucleolar dominance is observed in human–mouse somatic cell hybrids [20]. However, in the present study, we observe that in the F_1_ hybrids of BSB **×** TC the 45S rRNA genes of neither BSB nor TC are repressed. In the F_2_ hybrids, 45S rRNA genes derived from one progenitor are silenced in some individuals, thus showing nucleolar dominance; while there are also some individuals that express 45S rRNA genes derived from both parental species with no nucleolar dominance evident. These results indicate that nucleolar dominance patterns in the hybrid progeny of BSB and TC are different. Our results are partially consistent with nucleolar dominance research on allopolyploid *Arabidopsis*. In natural *Arabidopsis suecica*, an allotetraploid (amphidiploid) hybrid of *Arabidopsis thaliana* and *Cardaminopsis arenosa*, the *A. thaliana* rRNA genes are repressed. However, *A. thaliana* rRNA gene silencing is variable in synthetic *A. suecica* F_1_ hybrids. Two generations are needed for *A. thaliana* rRNA genes to be silenced in all lineages [17]. Variable rRNA gene silencing in *A. suecica* F_1_ hybrids suggests there is a stochastic component to the establishment of nucleolar dominance. Furthermore, completion of nucleolar dominance in the *A. suecica* F_2_ generation implies that silencing is cumulative and is not erased at meiosis. *De novo* cytosine methylation is a candidate DNA modification that might explain the stochastic onset and progressive establishment of nucleolar dominance [21–25] and is consistent with the depression of silenced rRNA genes by aza-dC (5-azadeoxycytidine). However, the number of generations needed to establish complete nucleolar dominance in hybrids of BSB × TC is unknown.

As reported [3], the nucleolar dominance is not random in genetic hybrids. Instead, it is always the rRNA genes from the same parent that are silenced. The choice mechanism is not based simply on the number of rRNA genes at an NOR, as NORs with fewer genes can be dominant over NORs with more rRNA genes. However, in this study, neither the set of 45S rRNA genes from BSB nor that from TC is consistently silenced in the F_2_ hybrid (Fig. 3). These results provide new insights into the phenomenon of nucleolar dominance in genetic hybrids. The question of why one set of 45S rRNA genes is not completely dominant over the other deserves further study.

There are 23.3% individuals in the F_2_ hybrids of BSB × TC only inherited the set of 45S rRNA genes of one progenitor (Table 1). This has been shown by both ITS1 fragment amplification and sequencing of 18S rDNA PCR products. Hybridization can result in alterations of in gene expression, chromosomal structure and genome size [26]. In the resynthesized hybrids and allopolyploids, loss of parental fragments and/or the appearance of novel fragments are commonly observed. Sequence elimination in the resynthesized allopolyploid wheat may account for a relatively large amount (~14%) of genome- or chromosomal-specific DNA sequences [27, 28]. In this study, all F1 hybrid individuals inherited the 45S rDNA ITS1 sequences of both parents, which demonstrates the genetic hybridity of the F_1_ offspring. Among F2 hybrids, 76.7% of the F_2_ hybrids inherited both parental types whereas 23.3% individuals lost the ribotype of one progenitor (BSB or TC) (Table 1), which indicates that 45S rDNA sequence elimination may have occurred in the F_2_ hybrids. These results support the theory that hybridization may lead to genetic change in the genomic DNA of the hybrids. We think that the genetics changes in the hybrids may have some relationship with the unique nucleolar dominance patterns in the hybrid lineage.

## Conclusions

Nucleolar dominance patterns in intergeneric hybrids of BSB and TC are distinctive. Nucleolar dominance is not established in F_1_ hybrids and is observed in some F_2_ hybrids, indicating that genetic changes occur gradually in different generations in the hybrid lineage. In addition, neither the set of 45S rRNA genes from *M. amblycephala* nor that from *C. alburnus* is consistently silenced in the F_2_ hybrids, revealing that the nucleolar dominance is random in the F_2_ hybrids of *M. amblycephala* and *C. alburnus*. These findings reveal new insights into the phenomenon of nucleolar dominance in genetics hybrids and provide a good model to study the molecular mechanism by which one progenitor’s genes are selectively silenced.

## Methods

### Study material and generation of hybrid populations

Individuals of BSB, TC, F_1_ and F_2_ hybrid of BSB × TC are obtained from the Engineering Research Center of Polyploid Fish Breeding and Reproduction of the State Education Ministry, China, located at Hunan Normal University. The formation of the F_1_ and F_2_ hybrid of BSB × TC is described in our previous paper [14].

### Detection of hybridization

To identify genetic markers to distinguish between BSB and TC, a pair of primers based on conserved regions of 18S and 5.8S ribosomal DNA (rDNA) in fish (P4F, 5′-AGTCGTAACAAGGTTT CCGTAG-3′; P4A, 5′-ATC(A/G)ATGTGTCCTGCAATTCAC-3′) are designed and synthesized to amplify 45S rDNA internal transcribed spacer 1 (ITS1) directly from genomic DNA. Total genomic DNA of BSB, TC, F_1_ and F_2_ hybrids of BSB × TC is extracted from liver tissue using a phenol/chloroform extraction method. PCR reactions are carried out in a volume of 50 μl and contained approximately 50 ng genomic DNA, 1.5 mM MgCl_2_, 250 mM each dNTP, 0.4 mM each primer, and 2.5 U HS Taq polymerase (TaKaRa, Dalian, China). The thermal program consists of an initial denaturation step at 94 °C for 5 min, followed by 30 cycles (94 °C for 30 s, 56 °C for 30 s, and 72 °C for 45 s) and a final extension step at 72 °C for 10 min. The PCR products are separated in a 2% agarose gel using TAE buffer. The DNA fragments are purified using a gel extraction kit (TaKaRa), an adenine residue is added to the end of the PCR products with DNA A-tailing kit (TaKaRa), and ligated into the pMD18-T vector (TaKaRa). The plasmids are transformed into *Escherichia coli* strain DH5a and purified, and the inserted DNA fragments are sequenced using an automated DNA sequencer (ABI PRISM 3730, Applied Biosystems, Carlsbad, CA, USA). To examine sequence homology and variation among the fragments amplified from BSB, TC, sequences are aligned using BioEdit [29] and Clustal W [30].

### Sequencing of 18S rDNA

To amplify the 18S rDNA fragment directly from genomic DNA of BSB, TC and their hybrids, a pair of primers based on those described by Singh et al. [31] (18S P1F, 5′-TTGGTGACTCTCGA TAACCTCGGGC-3′; 18S P1R, 5′-CCTTGTTACGACTTTTACTTCCTC-3′) are designed and synthesized. For sequencing, a second pair of primers (18S P2F, 5′-CTTGTCTCAA AGATTAAGCCATGC-3′; 18S P2R, 5′-CTGCTGCCTTCCTTGGATGTGGT-3′) based on the 45S sequence of common carp (GenBank, accession number: JN628435.1) are designed and synthesized to amplify the upstream sequences of 18S rRNA genes containing the single nucleotide polymorphism (SNP) site confirmed with the 18S P1F/P1R primer pair. PCR reactions are carried out in a volume of 50 μl and contained approximately 50 ng genomic DNA, 1.5 mM MgCl_2_, 250 mM each dNTP, 0.4 mM each primer, and 2.5 U HS Taq polymerase (TaKaRa). With the 18S P1F/P1R primer pair, the thermal program consist of an initial denaturation step at 94 °C for 5 min, followed by 30 cycles (94 °C for 30 s, 56 °C for 45 s, and 72 °C for 2 min) and a final extension step at 72 °C for 10 min. With the 18S P2F/P2R primer pair, the thermal program consist of an initial denaturation step at 94 °C for 5 min, followed by 30 cycles (94 °C for 30 s, 56 °C for 30 s, and 72 °C for 45 s) and a final extension step at 72 °C for 10 min. The PCR products are separated in a 1.5% agarose gel using TAE buffer. The DNA fragments are sequenced using the method described above.

### Detection of single nucleotide polymorphism and 45S rRNA expression

Detection of species-specific rRNA genes and transcripts in interspecific hybrids between BSB and TC is limited by the low level of interspecific divergence in the 18S rRNA genes. On the basis of the 18S rRNA gene sequences amplified by the 18S P2F/P2R primer pair, a SNP at position 308 of the 18S rRNA gene is fixed for cytosine in BSB (DDBJ accession number: AB860215) and thymine in TC (DDBJ accession number: AB860216).

Species-specific copies of rDNA and the corresponding rRNA transcripts are distinguished on the basis of the C308T SNP with direct sequencing of PCR products. Total RNA and genomic DNA are simultaneously isolated from single adult fish. 30 individuals of each kind of fish (including BSB, TC, F_1_ and F_2_ hybrids of BSB × TC) are selected at random. Total RNA is isolated from liver tissue of different individuals with the Total RNA Isolation System (Omega Bio-Tek, Norcross, GA, USA). DNA is subsequently extracted using the TRI-Reagent (Omega Bio-Tek, Norcross, GA, USA) extraction protocol from the same sample. RNA is treated with DNase I (Amplification Grade, Invitrogen, Carlsbad, CA, USA) and first-strand cDNA is synthesized with Superscript III (Invitrogen) using an 18S rRNA gene-specific primer (5′-TGCTGCCTTCCTTGGATGTG-3′). A 500 bp fragment containing the C308T polymorphism is amplified by PCR with the 18S P2F/P2A primer pair. The cycling profile consist of 95 °C for 30 s, 56 °C for 30 s, and 72 °C for 45 s for 30 cycles in a conventional thermal cycler. Identical conditions are used to amplify genomic DNA. PCR controls to detect contamination of RNA with genomic DNA are conducted for all samples. In these controls, DNase I-treated RNA is treated under the same conditions as the treatment samples, and only differed in that no transcriptase is added in the reverse transcription PCR (RT-PCR) process. Samples are considered free of contaminant DNA if PCR amplification failed. In some cases, contaminant genomic DNA is detected and a second DNase I treatment and RT-PCR are conducted. The PCR products are visualized in ethidium bromide-stained agarose gels and purified using a gel extraction kit (TaKaRa) [30].

## Additional files

Additional file 1: The alignment of 18S rDNA sequence of BSB and TC. The gap shows the single nucleotide polymorphism site between BSB and TC. (JPG 483 kb)
Additional file 2: The peak figures of the PCR products sequencing. A, The sequencing peak figures of the PCR products amplified from genomic and cDNA of 18S rRNA gene in BSB and TC. B, The sequencing peak figures of the PCR products amplified from genomic and cDNA of 18S rRNA gene in F_1_ hybrids of BSB × TC. C, The sequencing peak figures of the PCR products amplified from genomic and cDNA of 18S rRNA gene in F_2_ hybrids of BSB × TC. Only the SNP site and 2 upstream and downstream nucleotides are showed. Note: For every pattern of each kind of fish, one sample was used to present. (PPT 275 kb)

## Abbreviation

BSB: Blunt snout bream
NORs: Nucleolus Organizer Regions
TC: Topmouth culter
