# Supplementary figures and images for: Unique nucleolar dominance patterns in distant hybrid lineage derived from Megalobrama Amblycephala × Culter Alburnus
Source: BMC Genet. 2016 Dec 5;17:150. doi: 10.1186/s12863-016-0457-3 (PMC5139125; doi:10.1186/s12863-016-0457-3)

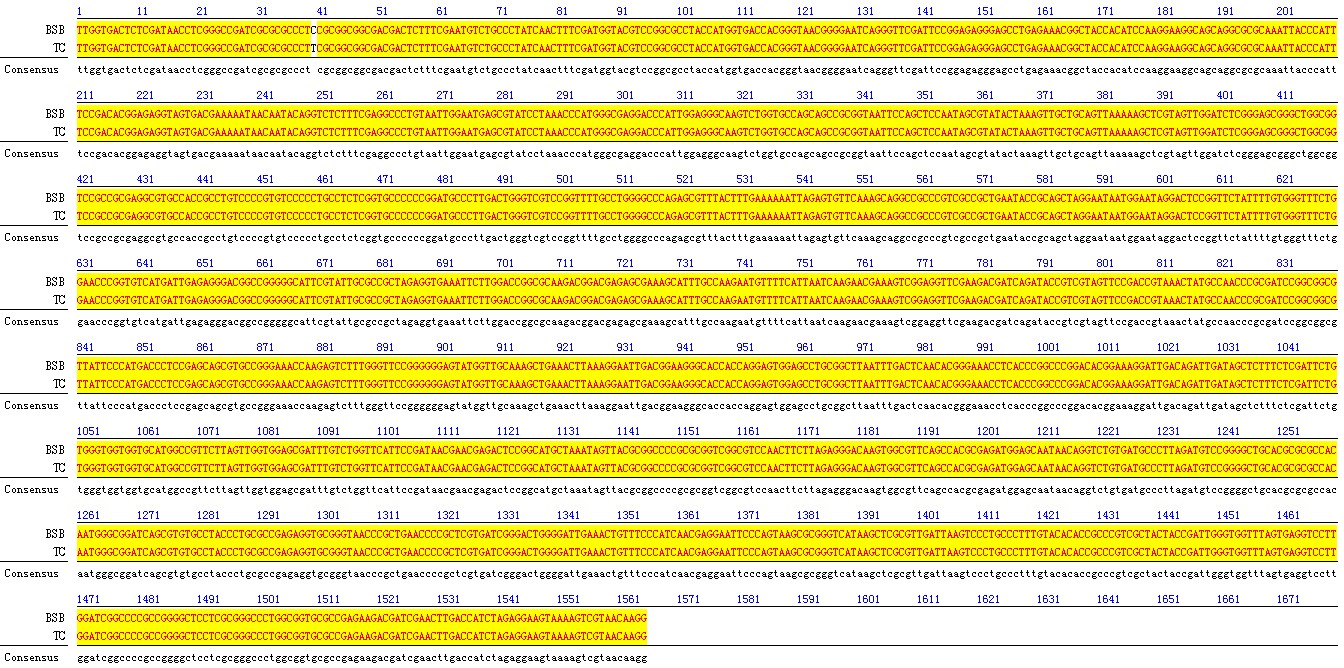

Supplement: Additional file 1: — The alignment of 18S rDNA sequence of BSB and TC. The gap shows the single nucleotide polymorphism site between BSB and TC. (JPG 483 kb) [file 12863_2016_457_MOESM1_ESM.jpg]
