# Supplementary material for: Unique nucleolar dominance patterns in distant hybrid lineage derived from Megalobrama Amblycephala × Culter Alburnus
Source: BMC Genet. 2016 Dec 5;17:150. doi: 10.1186/s12863-016-0457-3 (PMC5139125; doi:10.1186/s12863-016-0457-3)
Supplement: Additional file 2: — The peak figures of the PCR products sequencing. A, The sequencing peak figures of the PCR products amplified from genomic and cDNA of 18S rRNA gene in BSB and TC. B, The sequencing peak figures of the PCR products amplified from genomic and cDNA of 18S rRNA gene in F1 hybrids of BSB × TC. C, The sequencing peak figures of the PCR products amplified from genomic and cDNA of 18S rRNA gene in F2 hybrids of BSB × TC. Only the SNP site and 2 upstream and downstream nucleotides are showed. Note: For every pattern of each kind of fish, one sample was used to present. (PPT 275 kb) [file 12863_2016_457_MOESM2_ESM.ppt]

## Slide 1
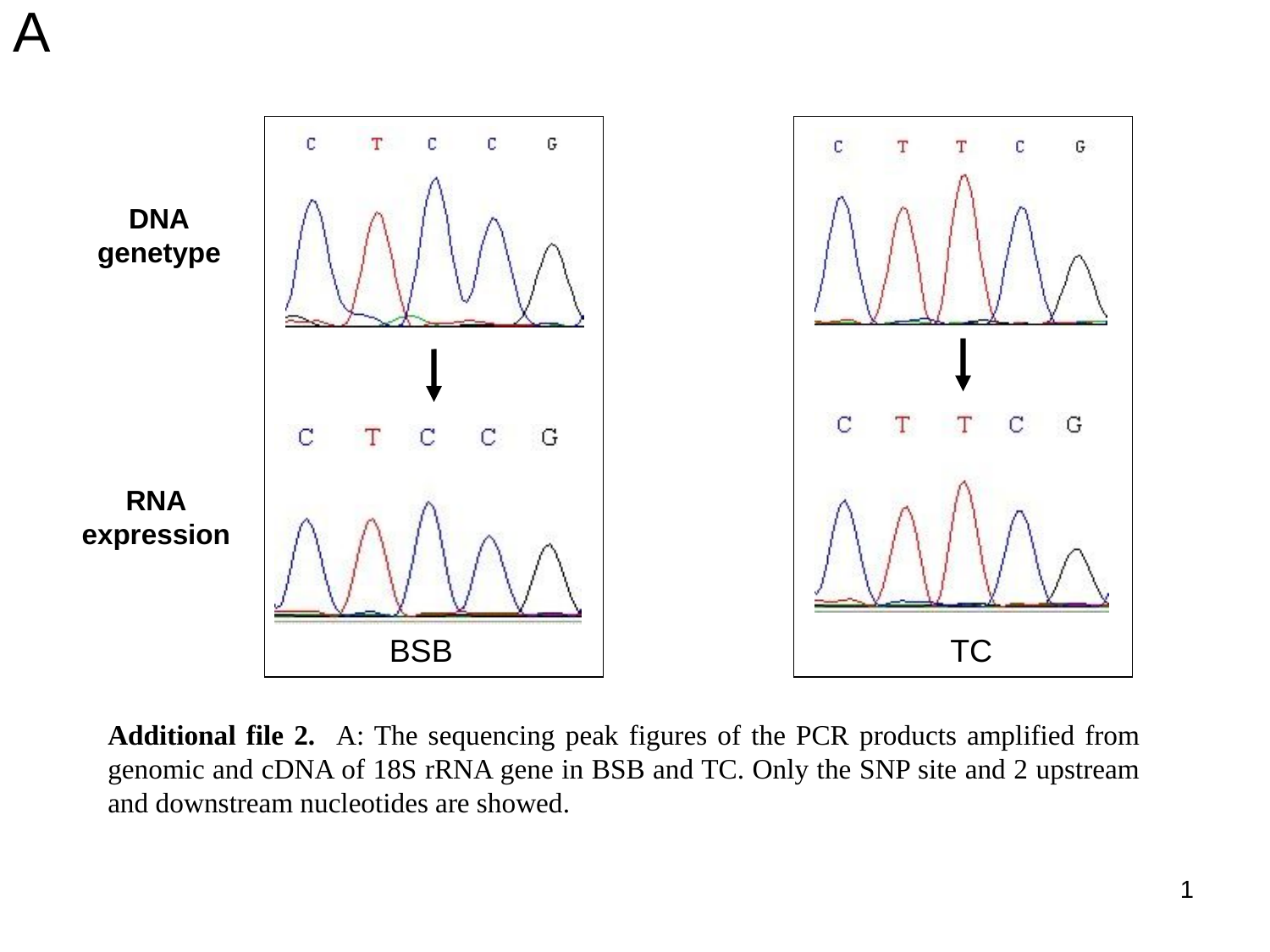

A
DNA
genetype
RNA
expression
BSB
TC
Additional file 2. A: The sequencing peak figures of the PCR products amplified from genomic and cDNA of 18S rRNA gene in BSB and TC. Only the SNP site and 2 upstream and downstream nucleotides are showed.
<number>

## Slide 2
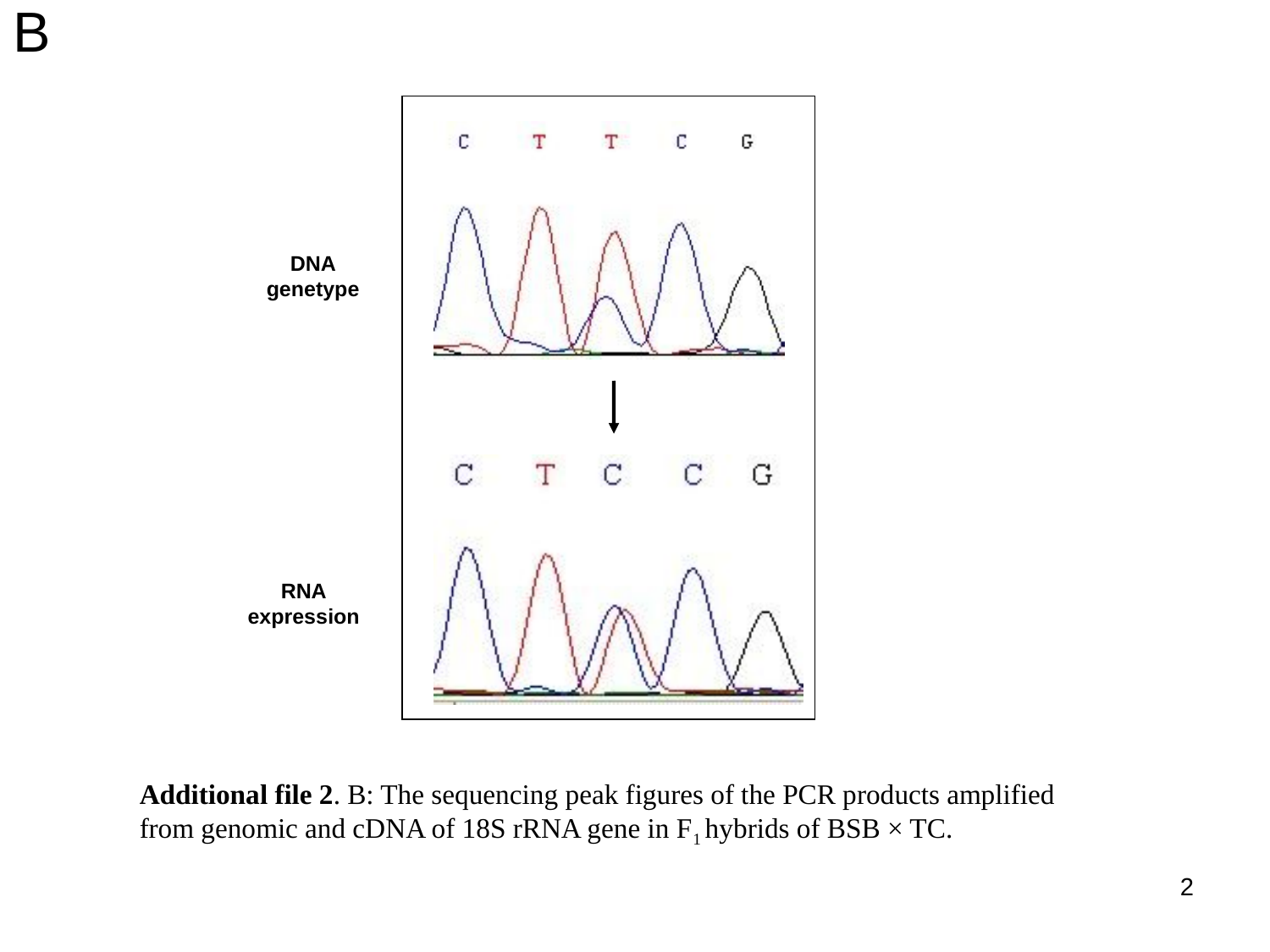

B
DNA
genetype
RNA
expression
Additional file 2. B: The sequencing peak figures of the PCR products amplified from genomic and cDNA of 18S rRNA gene in F1 hybrids of BSB × TC.
<number>

## Slide 3
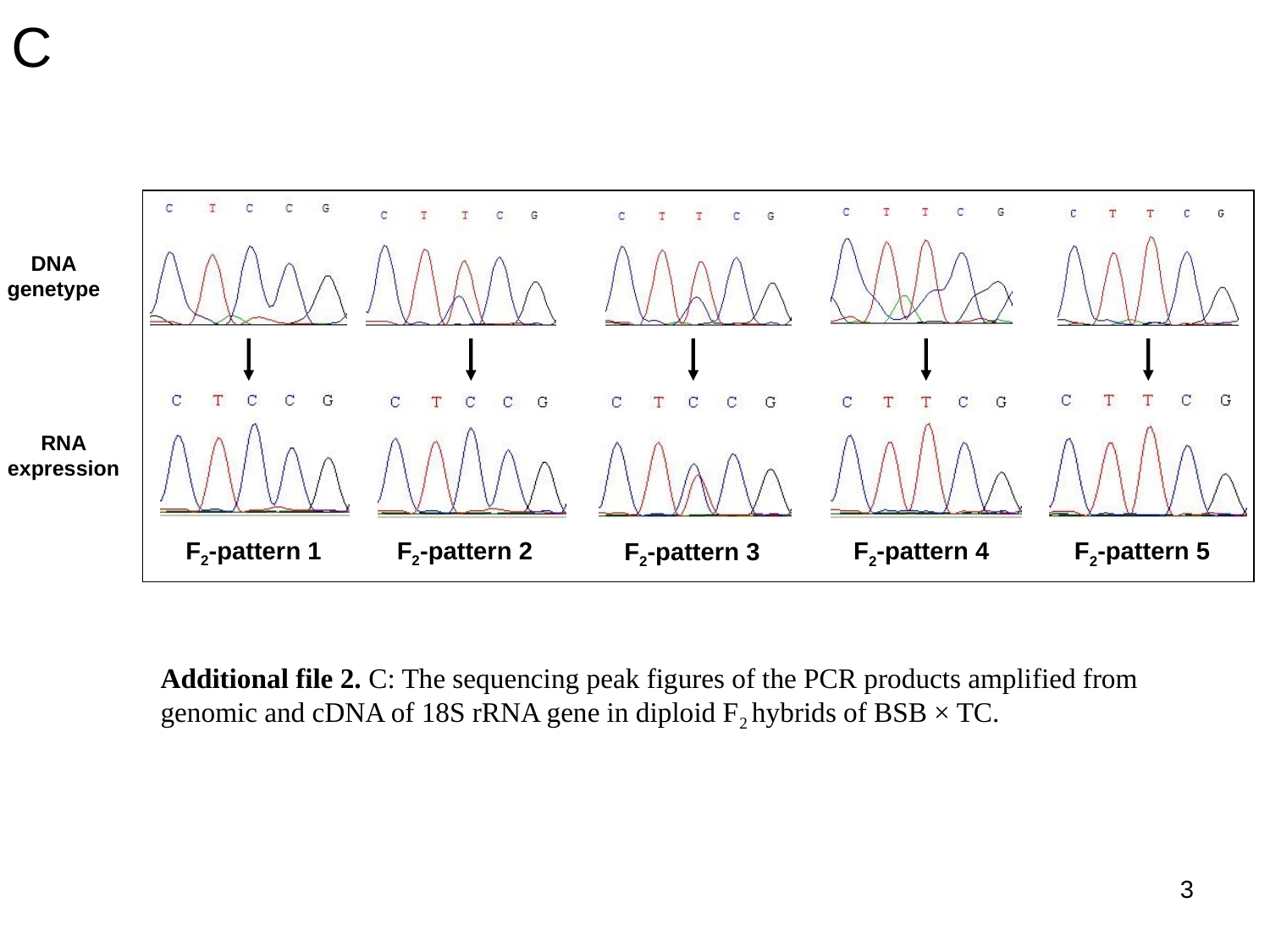

C
DNA
genetype
RNA
expression
F2-pattern 1
F2-pattern 2
F2-pattern 4
F2-pattern 5
F2-pattern 3
Additional file 2. C: The sequencing peak figures of the PCR products amplified from genomic and cDNA of 18S rRNA gene in diploid F2 hybrids of BSB × TC.
<number>
